# The Crawford Crown

**Published:** 1902-03-15

**Authors:** H. H. Johnson


### THE CRAWFORD CROWN.

There are now so many crowns that it seems almost superfluous to add another, but when we recall that we have not in all the number what might be termed an ideal bicuspid crown, I feel justified in citing an improvement. The Richmond bicuspid crown is strong and durable when well made, but has the objectional feature of a great display of gold about the occlusal surface, furthermore the facing is liable to be checked in soldering.

With the consent of Dr. J. Y. Crawford I herewith present one which should rightfully be termed the Crawford crown, and it will be found to be almost if not quite the ideal crown. Dr. Crawford has worked it up by embodying some of the old Richmond ideas with original ones of his own. It is made as follows—The root is prepared by grinding it off about even with the free margin of the gum, avoiding any oval or round shape at the end and removing any bulging shape at the sides. The canals are then enlarged for the reception of platinum posts, not forgetting to ream out with a large bur the orifices into a counter-sink. A band is carefully fitted about the circumference of the root, to which a flat top is soldered, forming a cap. This flat top is placed on the root and burnished down into the counter-sunk orifices of the pin holes. The holes for the pins are now punched at the indicated spots, the pins placed in position, the whole removed and the pins soldered in place from the upper side, filling the counter-sunk holes about the pins flush with solder. The cap and pins united are now placed back on the root and a bite and impression taken, from which an articulating model is made and mounted in an articulator.

The gold cap is now on the model which represents the mouth, and the pins are left projecting above the surface of the cap. Grind these off even with the top of the cap. The tooth is now selected, and it must be a vulcanite bicuspid. Adjust this to a proper occlusion by grinding off perfectly flat at the base, allowing it to fit flat against the top of the gold cap. Now with a strip of thick tinfoil take a measure of the circumference of the bicuspid tooth and make a gold band deep enough to reach to the grinding edge on the palatal side. Trim the band down to a narrow strip on the buccal side, press the tooth into this band and burnish the edges to a perfect adjustment. While the tooth is still inside it, grind or file off the base of the band flat and even with the base of the tooth. Remove the tooth and place the band, narrow edge front, on the gold cap and solder together with a minimum amount of solder. If the band fails to fill out to a proper contour on the palatal side, this deficiency may be remedied by thickening with solder at that point. After polishing the cap and band, the crown is completed by cementing the porcelain bicuspid firmly into place in the half cup which was soldered to the root cap. When this has been allowed to set hard out of the mouth it is ready to be cemented onto the root in the mouth and the job is completed.

We now have a very strong, natural looking crown, with a proper occlusion and showing a minimum amount of gold. The final result of the work is never in doubt from the begining, as the porcelain tooth never goes into the fire and there is no checking or other failure. This description of the method may appear somewhat voluminous, but it should not deter anyone from attempting to use it, as in practice it will be found not to take longer than the making of a Richmond crown, and in many ways it is more satisfactory.

—H. H. Johnson, in *Dental Digest.*

